# Surveillance along the Rio Grande during the 2020 Vesicular Stomatitis Outbreak Reveals Spatio-Temporal Dynamics of and Viral RNA Detection in Black Flies

**DOI:** 10.3390/pathogens10101264

**Published:** 2021-10-01

**Authors:** Katherine I. Young, Federico Valdez, Christina Vaquera, Carlos Campos, Lawrence Zhou, Helen K. Vessels, J. Kevin Moulton, Barbara S. Drolet, Paula Rozo-Lopez, Angela M. Pelzel-McCluskey, Debra C. Peters, Luis L. Rodriguez, Kathryn A. Hanley

**Affiliations:** 1 Department of Biology, New Mexico State University, Las Cruces, NM 88003, USA; fvaldez2988@gmail.com (F.V.); tina12@nmsu.edu (C.V.); campos73@nmsu.edu (C.C.); lzhou@nmsu.edu (L.Z.); khanley@nmsu.edu (K.A.H.); 2 Jornada Experimental Range Unit, Agricultural Research Service, US Department of Agriculture, Las Cruces, NM 88003, USA; debra.peters@osec.usda.gov; 3 Foreign Animal Disease Research Unit, Plum Island Animal Disease Center, Agricultural Research Service, US Department of Agriculture, Greenport, NY 11944, USA; luis.rodriguez@usda.gov; 4 The Arthropod Collection, Department of Entomology, Plant Pathology, and Weed Science, New Mexico State University, Las Cruces, NM 88003, USA; hvessels@nmsu.edu; 5 Department of Entomology and Plant Pathology, Institute of Agriculture, University of Tennessee, Knoxville, TN 37996, USA; jmoulton@utk.edu; 6 Arthropod-Borne Animal Diseases Research Unit, Center for Grain and Animal Health Research, Agricultural Research Service, United States Department of Agriculture, Manhattan, KS 66502, USA; barbara.drolet@usda.gov; 7 Department of Entomology, Kansas State University, Manhattan, KS 66506, USA; paularozo@ksu.edu; 8 Surveillance, Preparedness and Response Service, Animal and Plant Health Inspection Service, United States Department of Agriculture, Fort Collins, CO 80526, USA; angela.m.pelzel-mccluskey@usda.gov

**Keywords:** vesicular stomatitis virus, Simuliidae, New Mexico, vector, river, rhabdovirus, arbovirus

## Abstract

Vesicular stomatitis virus (VSV) emerges periodically from its focus of endemic transmission in southern Mexico to cause epizootics in livestock in the US. The ecology of VSV involves a diverse, but largely undefined, repertoire of potential reservoir hosts and invertebrate vectors. As part of a larger program to decipher VSV transmission, we conducted a study of the spatiotemporal dynamics of *Simulium* black flies, a known vector of VSV, along the Rio Grande in southern New Mexico, USA from March to December 2020. Serendipitously, the index case of VSV-Indiana (VSIV) in the USA in 2020 occurred at a central point of our study. Black flies appeared soon after the release of the Rio Grande’s water from an upstream dam in March 2020. Two-month and one-year lagged precipitation, maximum temperature, and vegetation greenness, measured as Normalized Difference Vegetation Index (NDVI), were associated with increased black fly abundance. We detected VSIV RNA in 11 pools comprising five black fly species using rRT-PCR; five pools yielded a VSIV sequence. To our knowledge, this is the first detection of VSV in the western US from vectors that were not collected on premises with infected domestic animals.

## 1. Introduction

Vesicular stomatitis virus (VSV, family Rhabdoviridae) is the agent of vesicular stomatitis (VS) in livestock and is an important agricultural pathogen [1,2]. The clinical signs of VS are indistinguishable from foot-and-mouth disease (FMD), with blisters and lesions forming predominately in and on the mouth, nostrils, tongue, coronary band, and teats [2,3]. Infection with VSV can result in acute febrile disease with stomatitis, lameness and depression in cattle, horses, and pigs. In dairy cattle there is significant loss of milk production, and in beef cattle and pigs significant weight loss [2,3]. Disease detection usually results in quarantines to animal movement, together resulting in substantial economic loss [4,5,6,7].

The two serotypes of VSV, New Jersey (VSNJV) and Indiana (VSIV), are maintained in endemic transmission in a region that spans southern Mexico to northern South America [1,2]. Since 1906, epizootics of VSV in domestic livestock in the US have occurred at approximately 5- to 10-year cycles, spreading north from the US-Mexico border states of Arizona (AZ), New Mexico (NM), and Texas (TX) through the Mountain West region [2,8,9,10,11,12]. Despite this long history of VSV epizootics in the US, there remain large gaps in our understanding of the transmission of VSV. 

VSV is characterized as an arthropod-borne virus but can also be transmitted mechanically via the contaminated mouth parts of insects, via direct contact among vertebrate hosts, and via fomites [1,13]. While there is substantial, albeit circumstantial, evidence that epizootics of VSV are initiated by vector-borne transmission, the generalist nature of the virus in arthropods has made it difficult to pinpoint vector species that play a major role in the initiation and maintenance of VSV epizootics in the western US. Insect vectors are believed be important for transmission because: (i) VSV outbreaks generally occur along waterways that provide habitat for vectors [14], (ii) disease presence is reduced on premises that utilize insect control measures [15], (iii) VSV epizootics in the endemic region are correlated with onset of precipitation and high abundance of vectors [16], (iv) virus-infected vectors are found in VS affected premises and (v) in the US, cases generally cease in the winter when vector populations die off, and can re-emerge in the late spring when vector populations increase in abundance [8,11]. Multiple methods for detecting virus or viral genome have been used to identify vectors potentially involved in the VSV transmission cycle. These methods have detected VSV or its genome in hematophagous dipterans including Simuliidae (black flies) [17,18,19,20,21], Psychodidae (sand flies) [22], Ceratopogonidae (biting midges) [20,23], and Culicidae (mosquito) vectors [24]; as well as non-hematophagus dipterans including houseflies, (Muscidae), root-maggot flies (Anthomyiidae), and eye gnats (Chloropidae) [17,19]. The majority of VSV detections in insects have occurred on farms and ranches during concurrent VSV outbreaks. Experimental work has demonstrated that species of *Simulium* black flies, *Aedes* mosquitoes, *Lutzomyia* sand flies, and *Culicoides* biting midges are biologically competent vectors. These findings are thoroughly reviewed by Rozo-Lopez et al. [13]. However, information regarding the role of vectors in the natural transmission cycle of VSV, particularly during viral emergence and spread, is lacking.

**Figure 1:**
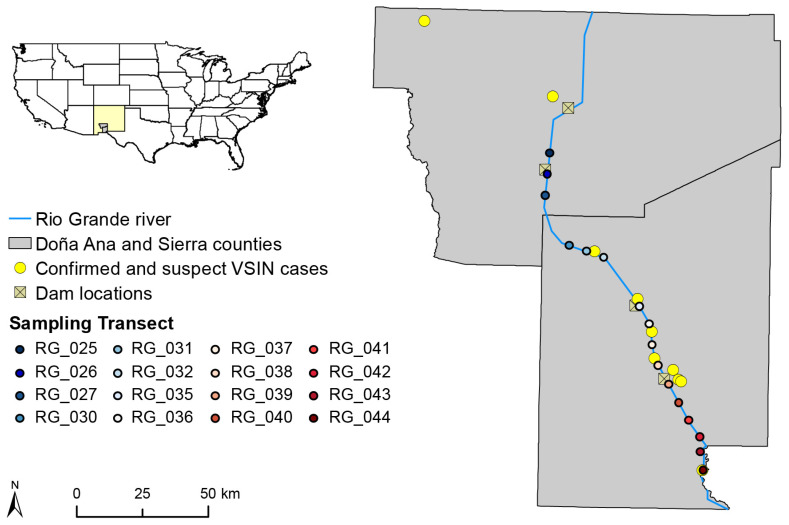
Study area along the Rio Grande within Doña Ana (south) and Sierra (north) counties in southern NM. The location of sampling transects along the Rio Grande are shown as well as the location of confirmed (by positive rRT-PCR and/or complement fixation test (CFT) with clinical signs present) and suspect (clinical signs present without diagnostic testing) VSIV cases from 2020 in these counties.

To begin to decipher the role of one of the major suspected VSV vectors in the US, we undertook a study of the spatiotemporal dynamics of black flies. Black flies play roles as pests and vectors of disease for humans and animals, but also act as ecological indicator species and ecosystem engineers [25,26,27]. Our study was conducted in the US state of New Mexico (Figure 1), which borders the Mexican state of Chihuahua. Since 1950, VS outbreaks have been documented in 17 different years in NM, five of which were caused by VSIV and 12 by VSNJV [9,10,12,28,29,30,31,32,33,34,35]. On 21 June 2019, APHIS reported a case of VSIV in Kinney Country, TX, representing the first case of VSV in the US since 2016 and the first case of VSIV in the US since 1998 [36]. The VSIV strain introduced in 2019 spread broadly across the mountain west, including NM [36]. The majority of VS cases in NM have been reported near the Rio Grande, the major waterway of this relatively arid state [14], suggesting transmission is driven by a vector that requires moving water for development, such as black flies [14,37]. 

Although we initially intended to collect black flies across the entire stretch of the Rio Grande in NM, COVID-19 restrictions limited us to the southern portion of the state. This more intense focus proved an unexpected opportunity, as the 2020 index case of VSIV in the US, reported on April 13, 2020, occurred in Las Cruces NM, the central point of our study region [38]. Thus, we were well poised to screen black flies for VSV that were collected over the same time period and within the same general area as VS cases were occurring. 

## 2. Results

### 2.1. Spatiotemporal Abundance of Black Flies and Relationship to Environmental Conditions 

**Figure 2:**
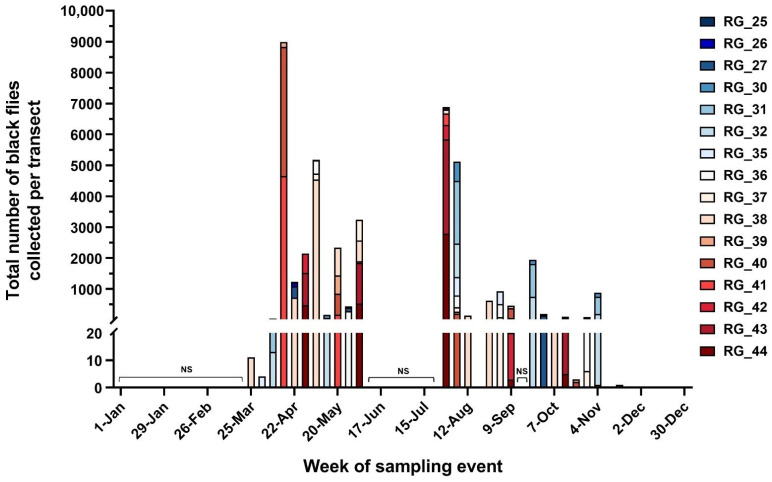
Total number of black flies collected per transect within a given sampling week from March 2020 to December 2020. Time periods when sampling did not occur are designated by “NS” brackets. North to south geographic distribution of transects along the Rio Grande are indicated as blue to red coloring, respectively. Each site, excepting RG_038, was sampled on five individual dates between April of 2020 to November of 2020 for a total of 25 trap nights across all sites. Site RG_038 was sampled approximately every two weeks from March of 2020 to December of 2020 for a total of 16 trap nights.

Black fly collections were conducted from 26 March 2020 to 17 December 2020 excepting two sampling pauses between 6 June to 29 July and between 12 September to 23 September. The first VS case in NM in this year was reported on 6 April 2020. A total of 40,699 black flies were collected from all sampling days between 26 March and 20 November, which was the last day on which black flies were found in traps in 2020. Black fly abundance at the transect level was highest in April, with an additional peak in late July (Figure 2). We regularly sampled one site, RG_038, during the course of the study to understand seasonal variation in black fly abundance. Our initial analysis of the four sampling sites in transect RG_038 showed substantial variation among sites within the transect and no significant difference in total blackfly abundance among sampling periods, omitting December collections (Repeated measures ANOVA: df = 2, *p* = 0.21). Nonetheless, visual inspection of mean abundance of black flies at RG_038 (Supplementary Figure S1) suggested substantial seasonal variation, with peaks in abundance in spring and late summer. Moreover, there was a significant difference in the total number of black flies per site collected across the months of the study (Negative Binomial GLM: df = 8, *p* = < 0.0001). Black fly abundance followed a seasonal trend with the late spring and summer months, April to August, having statistically similar and higher abundance than all other months, followed by September and November, and the months of March and October having the lowest black fly abundance (all comparisons based on least square means). Therefore, we decided to normalize counts at each site by dividing this count by the mean number of flies collected in the same month of sampling across all sites; this normalized value was used in analyses of abundance. 

There was a significant difference in the normalized number of black flies collected among transects (Negative Binomial GLM: df = 15, *p* = < 0.0001). Generally, normalized black fly abundance was fairly similar between transects but did vary from north to south along the Rio Grande. Site RG_031 had the highest normalized abundance and was statistically similar to a number of sites from the middle to the southern region of study area including RG_032, RG_035, RG_036, RG_038, RG_040, RG_041, RG_043, and RG_044 (Supplementary Figure S2). The two most northern sites, RG_025 and RG_026, had the lowest normalized black fly abundance and were statistically similar to sites that were also situated between the middle and southern portion of the Rio Grande including RG_030, RG_039, RG_037, and RG_042 (all comparisons based on least square means) (Supplementary Figure S2).

**Figure 3:**
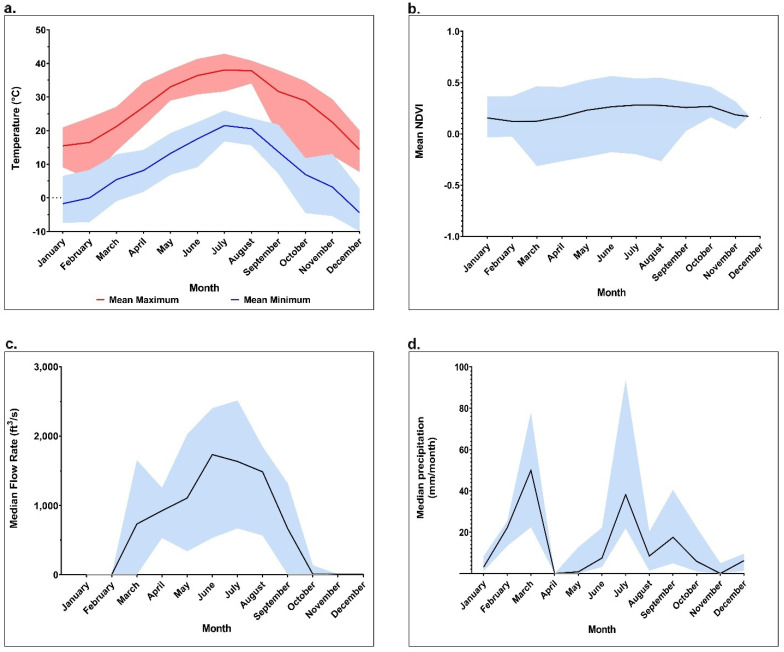
Central tendency and range (shaded region) of variables tested for associations with black fly abundance throughout 2020 across the transects sampled. (**a**) Monthly maximum and minimum temperature (°C) at each transect. (**b**) Monthly NDVI across all transects. (**c**) Monthly flow rate (ft^3^/s) along the Rio Grande from three gauges along the Rio Grande. (**d**) Total monthly precipitation (mm) at each transect.

To identify potential relationships between environmental and climatological conditions and black fly abundance we tested for differences in specific variables expected to impact black fly abundance and/or VSV occurrence, including vegetation greenness represented as Normalized Difference Vegetation Index (NDVI), river flow rate, maximum daily temperature, two-month lagged precipitation, and one-year lagged precipitation across the months of sampling. Maximum temperature, mean NDVI, and river flow rate per site all differed significantly across the months of the study (Kruskal–Wallis Tests, df = 8, *p* < 0.001 for all comparisons) (Figure 3). A post hoc analysis indicated that maximum temperature followed a standard progression of seasonal temperatures for the region, with the lowest maximum temperatures in March, April, and November and the highest maximum temperatures in the summer months of June, July, and August. Mean NDVI was lowest in March and highest in July across the sites sampled. However, NDVI varied greatly, with a range of 0.49 across the months sampled. Several of our sites occurred in city parks, where vegetation was regularly cleared, including, RG_031, RG_038, RG_039, RG_040, RG_043, and RG_044. 

In its southern reach in NM, the Rio Grande is only released seasonally, and mean flow rate of the river stayed statistically similar between March to July after water in the Caballo dam was released on 13 March 2020. Mean flow rate peaked in August with an average of 1421.3 (ft^3^/s), then immediately began decreasing in September until flow ceased on 26 September 2020. In southern NM, the Rio Grande runs through the Chihuahuan desert where rainfall is low and occurs primarily from July to October [39]. However, the 2020 monsoonal rainfall, which occurred predominately in late July and late August, was the weakest ever recorded [39]. Monthly precipitation was statistically similar and highest in March and July across the sites sampled. Other than an increase in precipitation in September, overall precipitation stayed relatively low between June and November with no rain in April. Total precipitation lagged by two months was statistically similar in March and April, steadily decreased to a low in June, rebounded until September and tapered off in October and November. One-year lagged precipitation stayed statistically similar from March to September, with March having the highest and November having the lowest yearly lagged precipitation.

To investigate the potential association of these variables with black fly abundance, we first tested correlation among variables. One year lagged precipitation and maximum temperature were significantly, positively correlated to all variables save that maximum temperature was negatively correlated with two-month lagged precipitation. Two month lagged precipitation was also negatively correlated to NDVI. Additionally, NDVI and flow rate had significant, positive correlations to one-year lagged precipitation (Supplementary Table S1). However, while there was significant correlation among variables, all relationships were relatively weak based on spearman’s rho; therefore, we included all variables together in subsequent analyses. Based on the overall seasonal trend of most environmental and climatological variables tested above, we analyzed abundance based on three periods: the early period was March to May, the middle period was June to August, and the late period was September to November. In the early time period, normalized black fly abundance increased significantly with increasing two-month lagged precipitation (Negative Binomial GLM: df = 112, *p* = < 0.0001) (Supplementary Table S2). During the middle time period, normalized black fly abundance increased with increasing maximum temperature (Negative Binomial GLM: df = 86, *p* = 0.02) (Supplementary Table S2). In the last three months of the study, black fly abundance increased with decreasing NDVI and increasing one-year lagged precipitation (Negative Binomial GLM: df = 134, *p* = < 0.0001) (Supplementary Table S2). 

### 2.2. Black Fly Identifications Using Molecular Barcoding

**Figure 4:**
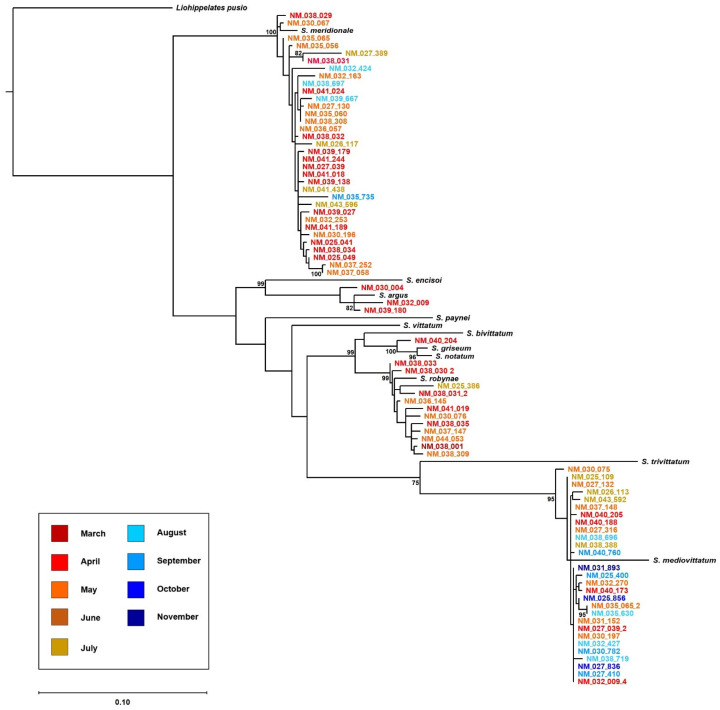
Maximum likelihood phylogeny (RAxML v.8, bootstrap values above 75 (1000 replicates) are noted at the nodes) of black fly pools collected in NM between March and November of 2020, 11 voucher specimen sequences, and one outgroup, *Liohippelates pusio*, inferred from mtDNA CoxI. NM black fly sequences are colored by the month in which they were collected and labeled with the state, the site, and the pool number. Nodes without bootstrap support indicate <70% bootstrap support and the scale bar represents 10% difference between two sequences. The location of collection for voucher specimens is provided in Table 1.

Although DNA barcoding using the cytochrome oxidase subunit I gene (CoxI) has been used to identify black flies to the species level in multiple studies across the world [40,41,42,43,44,45], there remains a general lack of available CoxI barcode reference sequences to perform species level analyses. Therefore, we created CoxI voucher barcodes for 11 morphologically confirmed *Simulium* species that were likely to occur along the lower portion of the Rio Grande in southern NM including *S. argus*, *S. bivittatum*, *S. encisoi*, *S. griseum*/*S. notatum*, *S. mediovittatum*, *S. meridionale*, *S. paynei*, *S. robynae*, *S. trivittatum*, and *S. vittatum* (Table 1). These barcodes were used to identify black fly pools to the species level using character-based phylogenetic analysis (maximum likelihood analysis) and distance-based methods (match between the query sequence and the reference database). We attempted to amplify CoxI from 119 pools of black flies initially categorized as morphogroups A (N = 53), B (N = 30), and C (N = 34) based on similar morphological characteristics including body color, leg color, and size, as well as two pools of black flies that were not characterized as one of these morphogroups (Supplementary Figure S3). Additionally, the selected pools also spanned the months when we detected black flies, March to November, and covered all transects over these months. Of these 119 pools, 20 (17%) failed to produce a CoxI sequence and were left as *Simulium* spp. and 99 (83%) were identified to species (Figure 4). There was no significant difference in the maximum daily temperature of collection for pools in which a CoxI barcode sequence was recovered and in which no barcode recovered (*t*-test: t = 1.62, df = 117, *p* = 0.11) (Supplementary Figure S4a). Five of the 11 voucher species were found in our collections, including *S. argus* (5% of identified pools), *S. griseum/S. notatum* (1%), *S. mediovittatum* (38.5%), *S. meridionale* (39.5%), and *S. robynae* (16%). Samples identified as the same species ranged between 97.3% to 99.4% sequence similarity to each other and 95.3 to 97.9 sequence similarity to voucher specimen sequences (Table 2). 

Only a small subset of collected pools were subjected to barcoding, so ecological inferences on the distribution of species are necessarily limited, and no analyses were conducted on species abundance. *S. argus* was only detected during the beginning of our study in April and seemed to be more geographically restricted to transects near the middle of our sampling region (Figure 4). The majority of these pools were collected in a heavily agricultural area near Hatch, NM. We detected one pool of *S. griseum/S. notatum*, which have been grouped together as they are potentially the same species with a sequence similarity of 97.7% and 97.2% to our *S. griseum* and *S. notatum* voucher sequences, respectively. This pool was collected early in the month of April from a southern site near the border with TX (Figure 4). *Simulium mediovittatum* was collected consistently from April until November of 2020 and collections were made from transects spanning the entire sampling portion of the Rio Grande (Figure 4). Similarly, *S. meridionale* was also detected along the entire sampling area; however, the latest it was detected was in September (Figure 4). *S. robynae* was detected in our first pool of black flies collected from the continuously sampled site RG_038 in March. *Simulium robynae* was primarily detected in April and May from transects located in the southern half our sampling area (Figure 4). However, *S. robynae* was detected from a single pool collected in July from our most northern site on the northern portion of the Caballo reservoir. All identified species were originally placed into at least two different morphogroup categories except the one *S. griseum/S. notatum* pool, which was put into the morphogroup B. Four of the five *S. argus* pools were categorized as morphogroup A, the other was placed in C. *Simulium mediovittatum* was predominately categorized as morphogroup C (N = 27); however, eight were categorized as B, two as A, and one as unknown. Of the 39 *S. meridionale* barcoded, 37 were described as morphogroup A and two as B. Similarly, 14 of the 16 *S. robynae* barcoded were described as morphogroup B and the other two as C. 

### 2.3. Detection of VSV RNA in Black Flies along the Rio Grande

A total of 77 black fly pools collected from March to May across all sites along the Rio Grande were screened for VSIV RNA, of which 57 (74%) were identified to species using barcodes (Table 3). These months covered the window of time when VSIV RNA was detected in horses in Doña Ana and Sierra counties during the outbreak (6 April 2020–20 May 2020) and included five black fly species and one group of unidentified black flies (*Simulium* spp.) VSIV RNA was detected in 11 pools comprising *S. mediovittatum*, *S. meridionale*, *S. notatum*/*griseum*, and *S. robynae* as well as unidentified black fly species (Table 3). The earliest detection of VSIV RNA was a pool collected on 17 April 2021.

Of the 11 pools where VSIV RNA was detected using rRT-PCR, we were able to amplify a VSIV sequence from five (38.5%) (Table 3, Figure S5). The rRT-PCR protocol used in this study was developed and thoroughly validated by the Canadian National Centre for Foreign Animal Disease in 2006 [46] and reviewed and re-validated in 2010 [47], during these validation experiments the limit of detection threshold was determined to be Ct ≤ 36. Days of collection of pools in which the virus failed to amplify were 2.6 °C hotter than days of collection for successful pools (*t*-test: t = 2.41, df = 11, *p* = 0.03) (Supplementary Figure S4b). Sanger sequencing for these five pools yielded a 160bp sequence within the L gene of VSV that was BLASTed against the NCBI database. These sequences shared a 94% to 99% sequence similarity to each other and an 82% to 87% similarity to a previously published VSIV sequence isolated from an infected cow in Wyoming during the 2019 Indiana outbreak (Genbank accession MT437283, accessed on 7 September 2021) (Supplementary Figure S5) [48]. There were 10 confirmed or suspect cases of VSIV in Doña Ana and Sierra counties in 2020 (Figure 1). These cases occurred in close proximity (2.1 to 10.7 km to closest case) to sampling locations where VSIV was detected in black flies collected along the Rio Grande (Figure 1). Virus isolation was attempted with the remaining VSIV RNA positive black fly pool homogenates by passaging in Vero cells, however these attempts were unsuccessful. Further efforts at isolation are ongoing.

## 3. Discussion

New Mexico has a long history of VSV epizootics and has played an important role as a steppingstone between introduction of the virus into the US and subsequent expansion through the mountain west. Much effort has gone into understanding the transmission dynamics of VSV on or near premises with a history of VSV transmission in NM [17,19,23,32,49,50,51] and other US states [32,52]. However, few studies have investigated the dynamics of potential VSV vectors at a fine scale within the outbreak range of the virus, with the exception of a detailed study of sandfly distributions in the southwestern US [53]. As part of a larger program that aims to elucidate the drivers of VSV incursion into the US, here we documented the spatiotemporal dynamics of a key vector group, black flies, along the major waterway in NM, the Rio Grande, during an ongoing VSIV outbreak in the state.

Our observations on black fly abundance support Adler et al.’s claim that flowing water is the lifeblood of black fly populations [27]. Black fly abundance increased steadily after the release of water from Caballo dam on 13 March 2020 into the stretch of the Rio Grande that flows through Doña Ana and Sierra counties, and abundance stayed high until the late summer when water flow began to decrease. However, even after the dam was closed on 26 September 2020, black flies were still consistently collected until the end of November, when temperatures dropped. The majority of these later collections occurred along the northernmost transects where flowing water can sometimes persist even after dam closure. There was significant variation in black fly abundance between transects. The two most northern transects, RG_025 and RG_026, had the lowest black fly abundance. This could be due to their proximity to two large reservoirs located just to their north as the size of a waterway can impact black fly utilization of a habitat [27,54]. Additionally, high water disturbance has been shown to impact Simuliidae abundance, which may explain the low abundance at RG_026 as it is located immediately south of the Caballo dam [55]. However, several other sites had statistically similar, low abundance without these river characteristics. Conversely, black fly abundance was highest at a centrally located transect, RG_031, which is situated just south of a large spillway. Therefore, an additional study focused on fine-scale river characteristics and adult black fly abundance would be useful in teasing out the determinants of the spatial variation we detected in southern NM. 

There were significant associations between environmental/climatological variables and black fly abundance in the early, middle and late trimesters of the study. Black fly abundance in the first sampling period of March to May increased with higher rainfall in the previous two months. Previous studies have demonstrated an association between precipitation regimes with increases in larval or adult black flies [56,57] as well as biting rates of black flies [57,58,59]. Our group and others have previously identified lagged precipitation as a driver of VS occurrence in NM and its expansion through the western US [8,49]. VSV was detected within our study area between April and May of 2020 as black fly abundance reached its initial peak. Of the over 40,600 black flies collected during our study, half were collected in these months. This finding is consistent with Hanson’s previous reports suggesting that seasonal and peak abundance of vectors is a likely driver of VSV transmission [29,60,61]. 

In the middle period of the study, black fly abundance increased with hotter maximum air temperatures. Both hotter [62,63] and cooler [58] air temperatures have been correlated with the abundance and host seeking behavior of adult black flies; additionally, in a flood-water system, larval growth rate of black flies increased with warmer air temperatures [64]. Furthermore, increased summer temperatures were associated with VS occurrence in 2005 [8]. In the last third of the study, September to November, black fly abundance increased with decreasing NDVI and increasing rainfall in the past year. Several of our transects occurred in parks and next to large agricultural fields where vegetation is cleared, possibly allowing faster warming of these areas as the weather cooled. It is particularly interesting that different environmental variables showed significant association with black fly abundance in different trimesters. This may reflect the natural progression of seasonal changes or shifts in the species composition of the black fly population over time. Similar variation in the association between black fly abundance and environmental variables across seasons has been reported by Jitklang et al. [65]. Unfortunately, as discussed below, we were not able to reliably identify black flies morphologically, nor did we do enough molecular barcoding to enable species-level analyses.

We have provided 99 new CoxI barcodes for five species of *Simulium*, further supporting the ability of the CoxI gene to differentiate individual species. Clear species identification within this group has great value, but morphological taxonomy is difficult due to small size and phenotypic similarity across species [27]. This issue was made clear when our attempt to categorize black flies into groups based on phenotypic characteristics failed to delineate between species confirmed by DNA barcoding. While DNA barcodes have successfully identified black flies to the species level [41], there remains a need to increase the number of available sequences. We recognize that our NM sampled black flies had between 95.3% to 97.9% sequence identity to our voucher sequences which is outside of the general cut off of <3% sequence divergence for species identification by DNA barcoding [66,67]. However, regression tree-based diagnostics visually identifies genetic similarity between sequences with additional statistical reliability and clearly indicates when results fail to match the vouchers used for diagnostics [68,69]. Thus, we used larger sequence divergence cutoffs due to the few closely related species sequences available and the larger overlap in intra- and interspecific genetic distances, as is the case for most insects [70,71]. We conclude that our NM samples with less than 6% sequence divergence to the voucher specimen sequences are likely conspecific because (1) most vouchers were collected elsewhere in the US, (2) there are few *Simulium* species barcodes available, and (3) we are confident these species were likely to occur in the southwestern US based on expert opinion. However, we note that the taxonomy of *S. griseum*/*S.notatum* is still in flux. Moreover, an additional species, *S. meyeri*, for which we did not have a voucher sequence, is closely related and phenotypically similar to both *S. griseum* and *S. notatum*.

Using these identification methods, we were able to report the first collection of *S. mediovittatum* outside of its previously described range in southern Texas [27]. The *Simulium* species collected in NM have varying distributions in the US. *S. argus* and *S. meridionale* are both broadly distributed, whereas *S. mediovittatum*, and *S. robynae* occur almost exclusively in the states bordering NM including AZ and TX [27]. Both *S. argus* and *S. mediovittatum* have also been collected in Mexico. Most of these species use streams in agricultural land, including irrigation canals, as habitat and both *S. argus* and *S. meridionale* have dispersal distances up to 30 km [27]. All five of the species collected have been described as frequent or sporadic pests of livestock, including horses and cattle [27]. Of the five *Simulium* species identified, *S. meridionale* is the best described and feeds on both avian and mammalian hosts [27]. *Simulium meridionale* has been known to voraciously feed upon and sometimes kill the nestlings of wild birds and has caused substantial economic impact on poultry farms [27]. *Simulium meridionale* has also been known to swarm feed on humans, causing hospitalization in some cases [27]. VSV infection and seroconversion has been detected in a broad range of vertebrate hosts [72,73,74,75,76]; however, viremia has rarely been demonstrated following experimental infection of vertebrate species and never reported from naturally infected animals, suggesting that an unknown reservoir maintains the virus in nature [1]. Given the generalist feeding habits of these five species of black flies, a study of host utilization via bloodmeal analysis could provide valuable insight into a potential vertebrate reservoir species. 

To our knowledge, we are making the first report of VSV detected in *Simulium* collected in habitats outside of farms and ranches with concurrent or historical epizootics of the virus. Additionally, this is the first detection of VSIV in *Simulium* in NM; we note that VSIV was also detected in 2020 from *S. meridionale* collected on a VSIV-confirmed premise in Kansas [20]. VSNJV has previously been detected in *S. vittatum* and *S. bivittatum* during outbreaks [17,21]. Of the five species in which VSIV was detected during our study, only *S. notatum* has been confirmed as a biological vector of VSNJV and VSIV experimentally [77]. The only other black fly species that has been shown to transmit VSV in the laboratory is *S. vittatum* [77,78,79]. 

The rRT-PCR assay used in this study was recently utilized to detect infection after experimental exposure of biting midges to VSV [80,81]; midges were considered positive for VSV with Ct values as high as 35.6 when orally infected, and 34.8 when intrathoracically infected. In the current study, the highest Ct value detected for a black fly deemed to be positive for VSV was 32.5. Of important note, previous work from others has shown that VSV RNA is able to persist in convalescent cattle as far as 5 months after inoculation, with no infectious virus ever being isolated successfully [82], suggesting the possibility that very low copy numbers of viral RNA could also persist in insects in the absence of replication-competent VSV. Our initial attempts at viral isolation from black flies in this study failed, but future attempts at viral isolation of VSV positive pools will be made at Plum Island Animal Disease Center. Another important caveat to our study is that we did not separate bloodfed black flies out of general pools, and so detection of viral RNA could simply reflect the presence of virus in undigested host bloodmeals. However, the recent detection of identical VSNJV sequences from black flies and infected horses on the same premise lends support to the notion that black flies play an important role in VSV transmission [21]. 

Given that NM has been a starting point for some previous VS epizootics, this study sheds substantial light on the potential role of black flies as vectors in the initial stages of VSV incursion into the US. We detected VSIV in black flies within 11 km and between 1 to 24 days to the closest confirmed or suspect case of VSV. Given this proximity, and despite the caveats discussed above, it is likely that at least some species of black flies were involved in VSV transmission to domestic animals during this outbreak. Interestingly, we were particularly successful in amplifying VSIV sequence from *S. mediovittatum*. This could have occurred if *S. mediovittatum* were more active on cooler days, as we did find that flies which yielded a positive PCR detection but not a sequence were collected on days that were almost 3 °C hotter than flies that yielded both a positive PCR and a sequence. However, it might also be explained if the VSIV titer in *S. mediovittatum* is higher than in other black fly species, although the limited Ct data from this study do not support that conclusion. Altogether, these findings warrant experimental tests of the ability of these black fly species, particularly *S. mediovittatum*, to biologically transmit the virus, which would help further deduce their roles in transmission during the 2020 outbreak in NM. Additionally, it is currently unknown how VSV might overwinter or be maintained in the environment in its outbreak range; a future study focused on detecting VSV in black flies prior to occurrence of cases in early spring is warranted.

## 4. Materials and Methods 

### 4.1. Study Location and Study Sites

The study was undertaken along the Rio Grande within Doña Ana and Sierra counties of southern NM (Figure 1). The Rio Grande is the principal river in NM, spanning the entire state from north to south, and providing black fly habitat for this arid region [27]. Water flow is managed along the Rio Grande and is regulated by 15 dams and 8 reservoirs. Due to regulations below Elephant Butte and Caballo reservoirs in the central part of the state, the water flow in the southern portion of the Rio Grande in Doña Ana and Sierra counties is seasonal, with water typically arriving in mid-March and ending in mid-October (https://www.ebid-nm.org/scada, accessed on 16 January 2021). A total of 16, 1 km-long transects were randomly distributed a minimum of 5 km apart along a 149 km-long stretch of the Rio Grande (ArcMap 10.6.1, ESRI, CA, USA) (Figure 1). Four collection sites were distributed within each transect approximately 250 m (N = 64 total sampling locations). 

### 4.2. Black Fly Collections 

Black flies were collected using either Centers for Disease Control (CDC) light traps with the light source disabled or EVS (Encephalitis Vector Survey) traps (Bioquip, CA, USA) from each of the four sites within each 1 km long transect. Due to the COVID-19 pandemic, it was not possible to obtain adequate numbers of either trap type as a single type for the study, but preliminary data showed no difference in efficacy between the two (see below). Both trap types were used with approximately two pounds of CO_2_ in the form of dry ice. Traps were hung from vegetation directly adjacent to the river no earlier than 15:00 and no later than 20:00 and collected no earlier than 08:00 and no later than 12:00 the following day. Each site, excepting RG_038, was sampled on five individual dates between April to November of 2020 for a total of 25 trap nights across all sites. Site RG_038 was sampled approximately every two weeks from March to December of 2020 for a total of 16 trap nights; data from this site was used to investigate seasonal variation in blackly abundance while holding habitat and position on the river constant. All sampling was paused between 6 June to 29 July and between 12 September to 8 October.

The success of black fly capture for CDC and EVS traps was compared at three transects. Two CDC traps and two EVS traps were randomly placed at the four sites within each of these transects and run for one sampling day. The mean number of black flies collected by trap type was compared using a paired *t*-test. There was no significant difference between the mean number of black flies collected between traps (df = 2, t = 1.97, *p* = 0.19) and therefore trap types were used equally throughout the study. 

Upon trap retrieval, trap bags were transported in a cooler, without ice, to prevent fly death. The entire catch from traps was immediately placed into a −80 °C freezer to prevent DNA and RNA degradation. Later, trap containers were removed from the freezer and black flies were sorted and counted on a chill table (Bioquip, California, USA). Morphological identification of black fly species is famously difficult, thus we sorted black flies into morphologically distinct groups (morphogroups) and later subjected key groups to molecular barcoding (see below). Black flies were then pooled into 1.5 mL Eppendorf tubes with no more than 20 individual flies, labeled with a morphogroups designation, date, and site of collection and stored again at −80 °C. All additional insects collected coincidentally during sampling were labeled as bycatch and saved in 15 mL falcon tubes at −80 °C for later analysis. 

### 4.3. Environmental Data Collection

Flowing water is known to be critical for black flies [27]; however, additional biotic and abiotic factors including air and water temperature, substrate type (vegetation or rocks), and elevation can affect black fly development, abundance, and diversity [27]. Additionally, our team has previously shown that vegetation greenness, precipitation, and maximum air temperatures are correlated with VSV occurrence in the US during previous outbreaks [8]. Therefore, we collected data on Rio Grande flow rates, temperature, precipitation, and NDVI at each site per transect for each collection period. River flow rates were recorded from the Elephant Butte Irrigation District data portal (https://www.ebid-nm.org/scada, accessed on 16 January 2021) between the times of trap placement and recovery of each sample. Daily maximum, minimum, and mean flow volume (m^3^/s) from the nearest river or dam station to the north of each transect was used to quantify flow rate; stations used included Elephant Butte dam, Caballo dam, Leasburg cable, and Mesilla dam.

Maximum and minimum daily temperature (°C) for the dates of sampling were obtained using Google Earth Engine from Daymet (https://daymet.ornl.gov/, accessed on 14 January 2021) at a 1 km × 1 km resolution. Two-month lagged and one-year lagged precipitation (mm) was also collected for each site from Daymet. For two-month lagged precipitation, the total rainfall from the previous two months was summed and for one-year lagged precipitation, the total rainfall from the given month in the previous year to the current year was summed. NDVI measurements were extracted from the Landsat 8 Collection 1 8-Day NDVI Composite dataset using Google Earth Engine at a 30 m resolution. These were combined in Google Earth Engine to generate shapefiles containing bi-monthly maximum, minimum, and mean NDVI data. Specific NDVI measurements for each site were then extracted in ArcMap 10.6.1 (ESRI, CA, USA).

### 4.4. Black Fly Morphological Identification

Each insect in the collection was first identified as a black fly or not a black fly based on descriptions given by Adler et al. [27]; black flies were then categorized into three morphogroups, designated A, B, and C, based on morphological characteristics including body color, leg color, and size. Five individuals from each morphogroups were pinned and high-definition photographs were taken. These images were then evaluated by an expert in Simuliidae systematics (co-author Dr. Kevin Moulton) and used to compared against 11 species that are known to occur in the southwest and near southern NM including *S. argus*, *S. bivittatum*, *S. encisoi*, *S. griseum/S. notatum*, *S. mediovittatum*, *S. meridionale*, *S. paynei*, *S. robbynae*, *S. trivittatum*, and *S. vittatum*. After initial investigation of the photographs, it was concluded that that morphogroups A was most likely *S. meridionale*, morphogroups B was likely *S. griseum* or *S. meyerae*, and morphogroups C was most likely *S. mediovittatum*. Subsamples of each were then subject to molecular barcoding (see below).

### 4.5. DNA and RNA Isolation from Black Flies

Black fly pools of no more than 20 individuals of the same morphogroups were homogenized using a motorized mortar and pestle until tissues appeared completely pulverized (approximately 1 min) on ice in 500 µL of cell culture media (MEM + 10% FBS, 1% non-essential amino acids, 1% penicillin/streptomycin and 5 µg/mL amphotericin B (all from Thermo-Fisher Scientific, Waltham, MA, USA), clarified by centrifugation at 4 °C for one minute at 12,000× *g*, and 250 µL of sample supernatant was moved to a clean tube and maintained on ice for RNA extraction and the remaining 250 µL was saved in a clean tube at −80 °C for potential virus isolation. Total RNA was extracted from 250 µL of the sample supernatant using TRIzol LS reagent (Invitrogen, Waltham, MA, USA) according to manufacturer’s instructions. RNA was precipitated from the first aqueous phase using isopropanol, washed twice with 75% ethanol, and resuspended in 50 µL of nuclease-free water. DNA was extracted from the reserved phenol phase of the TRIzol reaction by mixing with 400 µL of back-extraction buffer (BEB) [4 M guanidine thiocyanate, 50 mM sodium citrate, and 1 M Trizma base (all from Sigma-Aldrich, St. Louis, MO, USA)]. Samples were shaken by hand intensively for 30 s, then incubated for 10 min at room temperature, and centrifuged for 15 min at 12,000× *g* at 4 °C. The resulting aqueous phase was moved to a clean tube and DNA was precipitated using 400 µL ice-cold isopropanol. Samples were invert-mixed, incubated for 5 min at room temperature, and centrifuged for 15 min at 12,000× *g* at 4 °C, after which the supernatant was decanted off the pellet and the pellet was washed twice with 75% ethanol, and resuspended in 50 µL of nuclease-free water. RNA and DNA concentrations were determined using a Nanodrop spectrophotometer (Thermo-Fisher Scientific)

### 4.6. Black Fly Molecular Barcoding 

Despite there being an estimated 256 species of Simuliidae in the US, there is a lack of available barcodes to account for this diversity [27,83]. Therefore, to assess our morphological identifications, we extracted DNA from morphologically confirmed voucher specimens of 11 species common to the southwestern US held in the Moulton collection at University of Tennessee. Total DNA was extracted from these vouchers using Genomic DNA Clean & Concentrator-5 kit (Zymo Research, Irvine, CA, USA) according to the manufacturer’s protocol. PCR amplifications were conducted using GenePro (Bioer Technology Co., Hangzhou, China) thermal cyclers. CoxI fragments were amplified using TaKaRa Ex Taq Hotstart DNA polymerase (Takara Bio, Shiga, Japan) following the manufacturer’s suggested protocol, with 1.0 μL of template DNA and 3 μL each of 10 μM of custom forward (CoxI-F) and reverse (CoxI-R) primers within a 50 μL reaction volume (Supplementary Table S3). The following PCR regimen was used: 94 °C for 90 s; 5 cycles of 94 °C for 30 s, 56 °C for 30 s, and 72 °C for 120 s; 5 cycles of 94 °C for 30 s, 52 °C for 30 s, and 72 °C for 120 s; 9 cycles of 94 °C for 30 s, 48 °C for 30 s, and 72 °C for 120 s; and 30 cycles of 94 °C for 30 s, 44 °C for 30 s, and 72 °C for 120 s; cycling reactions were completed after a 5 min 72 °C incubation and held indefinitely at 15 °C until removed from instrument. Entire PCR reactions were electrophoresed in, and excised from, a 1% agarose gel run at 118V for 30 min. Amplicons were purified using Econospin® silica columns (Epoch Life Science, Missouri City, TX, USA). Purified PCR targets served as template for Sanger® sequencing in both directions in 20 μL reactions using 20-fold diluted (=0.4 μL) BigDye® v3.1 terminators (Applied Biosystems, Waltham, MA, USA) using a cycling regimen similar to above but with 47 (5 cycles), 45 (14 cycles), and 43 °C (40 cycles) touchdown annealing and 60 °C extensions. Sequencing reactions were cleaned using Centrisep columns (Princeton Separations, Freehold, NJ, USA) and dried in a Centrivap Concentrator (LABCONCO, Kansas City, MO, USA). Dried samples were sent to the University of Tennessee, Knoxville, Genomics Core for analysis using an ABI PRISM® 3100 Genetic Analyzer (Applied Biosystems). Sequences from opposing strands were reconciled and verified for accuracy using Sequencer 4.7 (Gene Codes Corp., Ann Arbor, MI, USA).

For black flies collected in NM, DNA extracted from black fly pools were subject to DNA barcoding using the universal CoxI primers LCO1490 and HC02198 [84] (Supplementary Table S3). PCR was performed using a total of 100 ng/µL template DNA in a 25 µL PCR reaction (1 µL of each primer, 1.5 µL of MgCl (50 mm), 2.5 µL of PCR buffer, 0.8 µL of dinucleotide triphosphates (dNTPs), 0.1 µL of Taq DNA polymerase (5 U/µL, reaction brought up to 25 µL in nuclease free water) and cycling conditions adapted from Rivera and Currie (2009) [40]. To confirm successful amplification of the PCR target, 4 µL of the PCR reaction was electrophoresed in 1.5% agarose gel at 100 V for 50 min then visualized on a GelDoc Go Imaging System (Bio-Rad, Hercules, CA, USA). The remaining 22 µL reaction for pools with confirmed PCR amplification was purified using the DNA Clean & Concentrator kit (Zymo Research) and sent to MCLAB (San Francisco, CA, USA) for sequencing on an ABI 3730XL sequencer (Applied Biosystems). Returned sequences were cleaned, trimmed and BLASTed against the NCBI database.

All generated sequences with a minimum length of 500 bp, including voucher specimen sequences and one outgroup sequence that matched *Liohippelates pusio* to 98.63% (Genbank Accession Number: HQ945300, accessed on 4th June 2021), were aligned using the MAFFT aligner in Geneious 2021.1 (Biomatters Ltd., Auckland, New Zealand) [85]. The barcoding region alignment, with no stop codons and gaps, was then used to reconstruct a phylogenetic tree to infer species identities based on their nearest closest ancestor to the voucher specimen sequences. When multiple identical sequences were detected from multiple pools at the same transect on the same date of sampling, one representative sequence was used in the phylogeny. GTR+I+G was inferred by ModelTest-NG [86,87] as the best evolutionary model according to Akaike information criterion (AIC). The best fit maximum likelihood tree was then inferred using RAxML version 8 [88]. Rapid bootstrapping was also conducted with 1000 bootstrap replicates. All black fly CoxI sequences are publicly available in BOLD (https://v4.boldsystems.org/, accessed on 5 July 2021) under the project title Simuliidae of New Mexico.

### 4.7. VSV Screening in Black Flies

Total RNA from select black fly pools was used to perform initial screening for VSV using rRT-PCR targeting the L gene of VSIV using TaqMan Fast Virus 1-Step MasterMix (Applied Biosystems, Waltham, MA, USA) and the CFX Connect Real-Time PCR Detection System (Bio-Rad) as previously described [47]. Primers and probe used were forward primer VSIV-F, VSIV-R, and VSIV probe (Supplementary Table S3). For amplification, the following conditions were used: 1 cycle of 50 °C for 30 min, 1 cycle of 95 °C for 20 s, and 40 cycles of 95 °C for 15 and 60 °C for 60 s. All PCR reactions included a positive control consisting of RNA isolated from VSIV (VP-98F strain), which was obtained from the World Reference Center for Emerging Viruses and Arboviruses (Galveston, TX, USA), as well as a no template control. To determine RT-qPCR efficiency, ten-fold serial dilutions of VSIV RNA (VP-98F) ranging from undiluted to 10^−7^ were used to construct a standard curve with the following efficiency parameters: y = −3.360× +10.877, R^2^ = 0.999, efficiency = 98.4%. RT-qPCR reactions with a Ct ≤ 36 were considered positive for VSIV RNA. For sequencing confirmation of VSV positive pools, 100 ng of RNA were used to perform cDNA synthesis using the SuperScript III cDNA synthesis kit (Thermo-Fisher Scientific) according to manufacturer’s instructions. Primers used for cDNA synthesis were the same as described above (VSIV-F and VSIV-R). PCR cleanup was performed on all reactions using the Roche High Pure PCR product purification kit (Millipore Sigma, Burlington, MA, USA) and cDNAs were sent to MCLABfor sequencing.

### 4.8. Statistical Analyses

A generalized linear model (GLM) with negative binomial distribution was used to test for differences the number of black flies collected across the normalized number of black flies across transects. months sampled and the. Differences between environmental and climatological variables including maximum temperature, mean NDVI, mean river flow rate, total monthly precipitation, two-month lagged precipitation, and one-year lagged precipitation, were tested across the months sampled using a Wilcoxon test and multiple comparisons using a Steel-Dwass all pairs test in JMP version 16 (SAS Institute Inc., Cary, NC, USA). Spearman’s correlation was used to test for correlation between climatological and environmental variables including maximum temperature, mean NDVI, mean river flow rate, total monthly precipitation, two-month lagged precipitation, and one-year lagged precipitation, collected at the day of sampling from each site. While there was significant correlation across variables, Spearman’s rho was low in all cases; therefore, all variables were included in the above-described models for additional models. A GLM with negative binomial distribution was also used to test for correlations between the normalized number of black flies and environmental and climatological variables in three different time periods: early, March to May, middle, June to August, and late, September to November. All negative binomial GLM models and correlations analysis were run in SAS version 9.4 (SAS Institute Inc.). 

## Figures and Tables

**Table 1:** Location of collection of *Simulium* voucher specimens used for CoxI barcoding.

Species Name	State	County	Location
*S. argus*	AZ	Pima County	Arivaca Creek
*S. bivittatum*	AZ	Pima County	Cienaga Creek
*S. encisoi*	AZ	Pima County	Arivaca Creek
*S. griseum*	NM	Valencia County	Belen irrigation canal
*S. mediovittatum*	TX	Kinney County	Pinto Creek
*S. meridionale*	NM	Doña Ana County	Rio Grande
*S. notatum*	AZ	Yavapai County	Santa Maria River
*S. paynei*	AZ	Pima County	Arivaca Creek
*S. robynae*	TX	Brewster County	Rio Grande
*S. trivittatum*	TX	Kinney County	Pinto Creek
*S. vittatum*	AZ	Pima County	Cienaga Creek

**Table 2:** Percent identity of southern NM black fly sequences identified as a designated species to voucher sequences of that species and between samples.

	*S. argus*		*S. meridionale*		*S. mediovittatum*		*S. robynae*	
	To Voucher Sequence	Between Samples	To Voucher Sequence	Between Samples	To Voucher Sequence	Between Samples	To Voucher Sequence	Between Samples
Max	97.60%	100%	98.90%	100%	97.80%	100%	97.40%	99.70%
Min	95.90%	96.50%	96.30%	95.20%	94.30%	98.10%	95.60%	95.20%
Avg	96.70%	97.80%	97.90%	98.50%	95.40%	99.40%	96.90%	98.10%
±SD	0.83%	1.05%	0.57%	1.02%	0.88%	0.37%	0.42%	0.97%

**Table 3:** VSIV detections in black fly pools screened using rRT-PCR and results of Sanger sequencing.

	March 2020				April 2020				May 2020			
Black Fly Species	Pools Screened ^1^	rRT-PCR Pos.	Ct Range	Sequences Amplified	Pools Screened	rRT-PCR Pos.	Ct Range	Sequences Amplified	Pools Screened	rRT-PCR Pos.	Ct Range	Sequences Amplified
*Simulium* sp.	−	−	−	−	8	0	−	0	13	2 (15.4%)	30.1–32.5	0
*S. argus*	−	−	−	−	3	0	−	0	−	−	−	−
*S. mediovittatum*	−	−	−	−	5	3 (60%)	30.1–30.4	2 (66.7%)	8	3 (37.5%)	30.1–31.6	2 (66.7%)
*S. meridionale*	−	−	−	−	14	0	−	0	12	1 (8.3%)	31.5	0
*S. notatum*/ *griseum*	−	−	−	−	1	1 (100%)	29.82	1 (100%)	−	−	−	−
*S. robynae*	1	0	−	0	3	0	−	0	9	1 (11.1%)	27.0	0

^1^ Pool size ranged from 1 to 20 black flies. Positive pools were collected from six transects: RG_027, RG_030, RG_031, RG_032, RG_038, RG_040.

## Data Availability

The data presented in this study are available upon request from the corresponding author. CoxI sequences for black flies are available in BOLD under the project SIMNM Simuliidae of New Mexico.

## Supplementary Materials

The following are available online at https://www.mdpi.com/article/10.3390/pathogens10101264/s1, Figure S1: Mean number of black flies at site RG_038, Figure S2: Mean normalized number of black flies between transects, Figure S3: Images of morpho group categories, Figure S4a: Temperature and black fly barcodes, Figure S4b: Temperature and VSIV detection, Figure S5: VSIV sequence alignment Table S1: Correlation matrix of environmental variables, Table S2: Model output for season black fly abundance and environmental variables, Table S3: Primer and probes used in study.
