# Supplementary material for: Surveillance along the Rio Grande during the 2020 Vesicular Stomatitis Outbreak Reveals Spatio-Temporal Dynamics of and Viral RNA Detection in Black Flies
Source: Pathogens. 2021 Oct 1;10(10):1264. doi: 10.3390/pathogens10101264 (PMC8541391; doi:10.3390/pathogens10101264)
Supplement: Supplementary file 1 [file pathogens-10-01264-s001.zip › pathogens-1338567-supplementary.pdf]

Supplementary Information for

Surveillance Along the Rio Grande During the 2020 Vesicular Stomatitis Outbreak Reveals Spatio-temporal Dynamics of and Viral RNA Detection in Black Flies

Katherine I. Young[1,2*†], Federico Valdez[1,3†], Christina Vaquera[1], Carlos Campos[1], Lawrence Zhou[1], Helen K. Vessels[4], J. Kevin Moulton[5], Barbara S. Drolet[6], Paula Rozo-Lopez[7], Angela M. Pelzel-McCluskey[8], Debra C. Peters[2], Luis L. Rodriguez[3], Kathryn A. Hanley[1]

[1] Department of Biology, New Mexico State University, Las Cruces, NM; fvaldez2988@gmail.com (F.V.); tina12@nmsu.edu (C.V.); campos73@nmsu.edu (C.C.); lzhou@nmsu.edu (L.Z.); khanley@nmsu.edu (K.A.H.)

[2] Jornada Experimental Range Unit, Agricultural Research Service, US Department of Agriculture, Las Cruces, NM; debra.peters@osec.usda.gov (D.C.P.)

[3] Foreign Animal Disease Research Unit, Plum Island Animal Disease Center, Agricultural Research Service, US Department of Agriculture, Greenport, NY; luis.rodriguez@usda.gov (L.L.R.)

[4] The Arthropod Collection, Department of Entomology, Plant Pathology, and Weed Science, New Mexico State University, Las Cruces, NM; hvessels@nmsu.edu (H.K.V.)

[5] Institute of Agriculture, Department of Entomology and Plant Pathology, University of Tennessee, Knoxville, TN; jmoulton@utk.edu (J.K.M.)

[6] Arthropod-Borne Animal Disease Research Unity, Center for Grain and Animal Health Research, Agricultural Research Service, US Department of Agriculture, Manhattan, KS; barbara.drolet@usda.gov (B.S.D.)

[7] Department of Entomology, Kansas State University, Manhattan, KS; paularozo@ksu.edu (P.R-L.)

[8] Surveillance, Preparedness and Response Service, Animal and Plant Health Inspection Service, US Department of Agriculture, Fort Collins, CO; angela.m.pezel-mccluskey@usda.gov (AMP-M)

† Joint first authors

Corresponding author: Katherine I. Young
Email: kiy761@nmsu.edu

This PDF file includes:

Figure S1 – Figure S5
Table S1 – Table S3

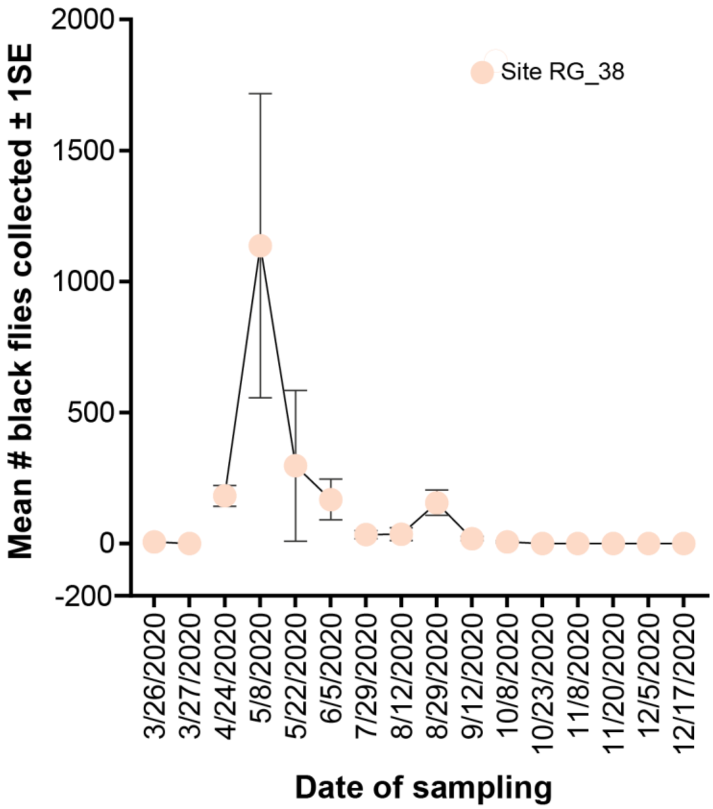

Figure S1. Mean number of black flies ± 1SE collected on each sampling date at a continuously sampled site, RG_38. Site RG_38 was sampled approximately every two weeks except for three longer breaks that are indicated by gaps in the line connecting points.

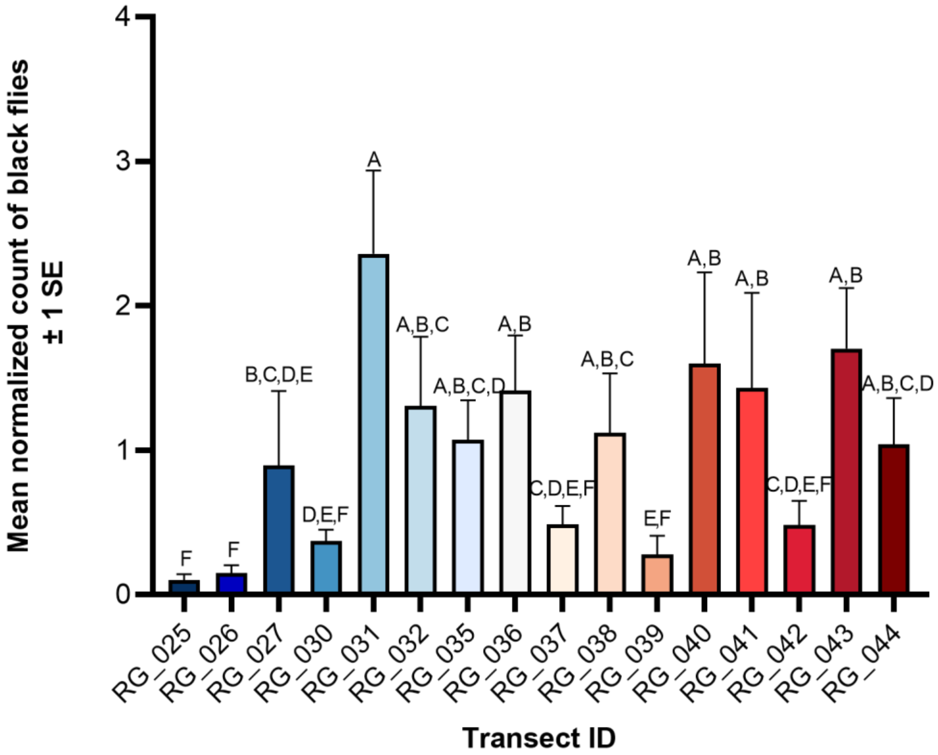

Figure S2. Mean normalized number of black flies ± 1SE collected at each transect along the Rio Grande (Negative-binomial GLM: df = 8, p = <0.001). Differences between individual transects are specified by letters above each column (based on least-square means). Columns with the same letters are not statistically different.

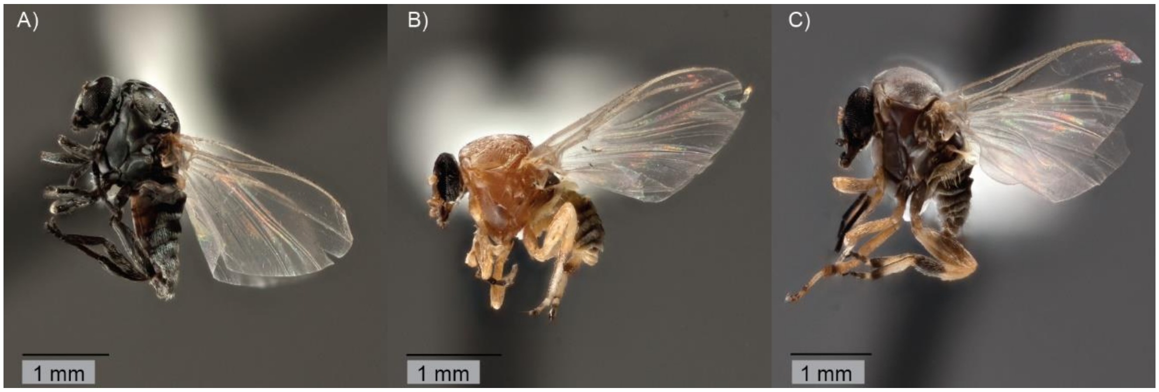

Figure S3. Representative images of the three morphogroup categories, A, B, and C, for black fly identification.

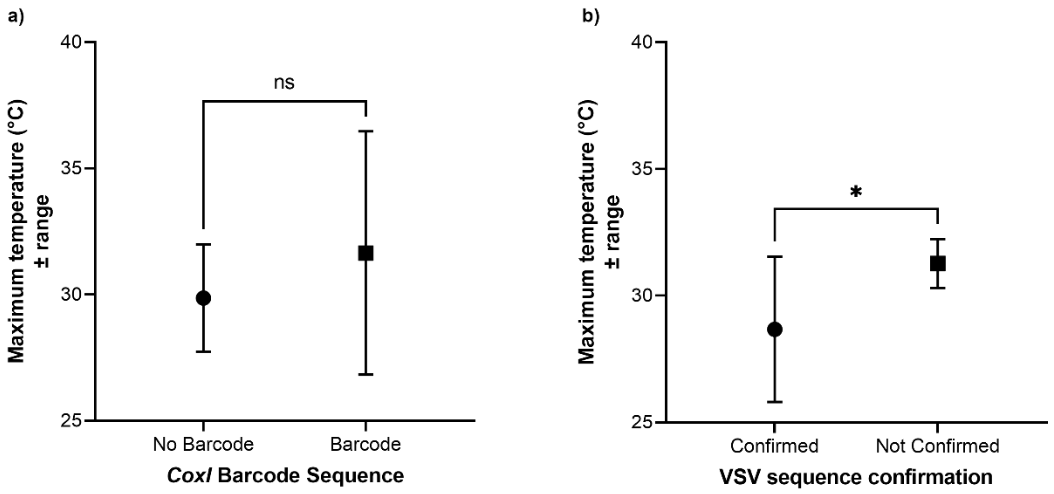

Figure. S4. Relationship between maximum temperature on the day of black fly collection and CoxI barcode and VSV sequence acquisition. **a)** Mean maximum temperature and maximum temperature range on the day of black fly collection for pools with CoxI barcode sequence confirmation and those without sequence confirmation. There was no difference in the maximum temperature with pools that did not produce CoxI barcode sequence and those that did (t-test: $t = 1.62$, $df = 117$, $p = 0.11$). **b)** Mean maximum temperature and maximum temperature range on the day of black fly collection for pools with sequence confirmation of VSIV and those without sequence confirmation. There was a significant difference in the maximum temperature with pools that did not produce a VSIV sequence being hotter than those that did (t-test: $t = 2.41$, $df = 11$, $p = 0.03$).

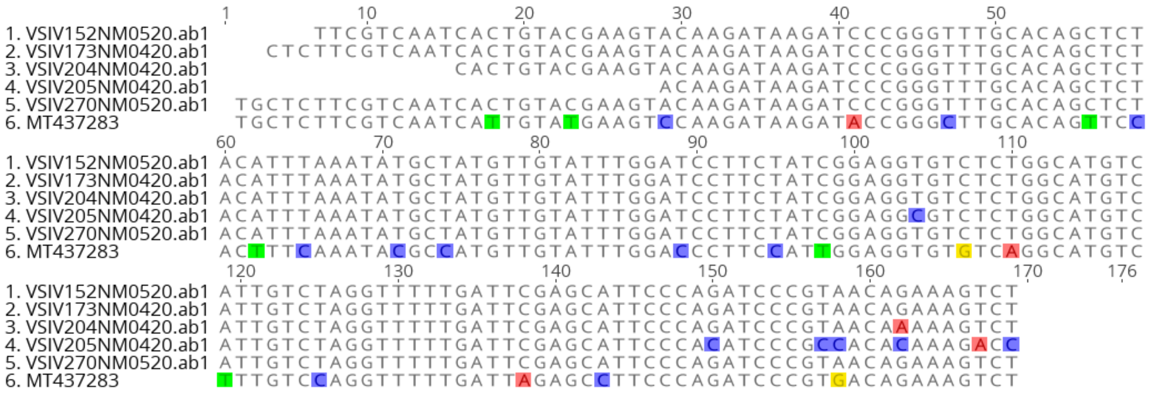

Figure. S5. Alignment of VSIV sequences of positions 7,288 to 7,456 (numbered from the first nucleotide in the genome) within the L gene obtained from black fly pools collected in Southern NM (sequence names starting with VSIV) in 2020 and a VSIV published sequence from an infected cow in Wyoming during the 2019 outbreak (MT437283).

Table S1: Correlation matrix based on Spearman's rho. Spearman rho for each combination of variables is shown on the bottom of the matrix and the p-value for each correlation is shown on the top of the matrix. P-values indicating significant correlation is highlighted in bold.

	**NDVI**	**Max temperature**	**Flow rate**	**2- month lagged precipitation**	**1-year lagged precipitation**
**NDVI**		**<0.001***	0.48	**<0.001***	**0.004***
**Max temperature**	0.22		<**0.0001***	**<0.001***	**<0.001***
**Flow rate**	0.04	0.34		0.14	**<0.001***
**2- month lagged precipitation**	-0.20	-0.20	-0.08		**<0.001***
**1-year lagged precipitation**	0.15	0.22	0.25	0.42	

Table S2: Model output for three negative-binomial GLMs run on normalized black fly and six predictor variables. Significant correlations are indicated in bold.

	**Early time period: March to May**			**Middle time period: June to August**			**Late time period September to November**		
**Predictor**	**Parameter estimate**	**DF**	**P-value**	**Parameter estimate**	**DF**	**P-value**	**Parameter estimate**	**DF**	**P-value**
Intercept	-1.3477	112	0.785	-5.1653	86	0.118	**-3.6582**	**134**	**0.0036**
Mean NDVI	-2.1817	112	0.051	-0.7166	86	0.479	**-6.8087**	**134**	**<0.001***
Average flow rate	0.0007	112	0.366	-0.00048	86	0.208	-0.00064	134	0.0772
Maximum temperature	0.04789	112	0.446	**0.1601**	**86**	**0.043**	0.04996	134	0.0823
Two-month lagged precipitation	**0.0615**	**112**	**<0.001***	0.01007	86	0.232	-0.01254	134	0.3148
One-year lagged precipitation	-0.0149	112	0.200	-0.00199	86	0.779	**0.01682**	**134**	**0.0120**

Table S3: Primer and probe sequences used in molecular analyses for black fly barcoding and VSV-IN detection.

Primer	Sequences (5′ to 3′)	Target Gene
CoxI-F	5′-ACAAAYCAYAARGATATYGG-3′	CoxI
CoxI-R	5′-YTARRTTWGMTCATGTTGMCAT-3′	CoxI
LCO1490	5′-GGTCAACAAATCATAAAGATATTGG-3′	CoxI
HC02198	5′-TAAACTTCAGGGTGACCAAAAAATCA-3	CoxI
VSIN-F	5′TGATACAGTACAATTATTTTGGGAC-3′	VSIN L gene
VSIN-R	5′-GAGACTTTCTGTTACGGGATCT-3′	VSIN L gene
VSIN-probe	FAM-ATGATGCATGATCCAGC-BHQplus	VSIN L gene
